# The 3′ UTRs of Brain-Derived Neurotrophic Factor Transcripts Differentially Regulate the Dendritic Arbor

**DOI:** 10.3389/fncel.2018.00060

**Published:** 2018-03-07

**Authors:** Kate M. O’Neill, Katherine E. Donohue, Anton Omelchenko, Bonnie L. Firestein

**Affiliations:** ^1^Department of Cell Biology and Neuroscience, Rutgers University, The State University of New Jersey, Piscataway, NJ, United States; ^2^Graduate Program in Biomedical Engineering, Rutgers University, The State University of New Jersey, Piscataway, NJ, United States; ^3^Graduate Program in Neuroscience, Rutgers University, The State University of New Jersey, Piscataway, NJ, United States; ^4^Biomedical Engineering Graduate Faculty, Rutgers University, The State University of New Jersey, Piscataway, NJ, United States; ^5^Neuroscience Graduate Faculty, Rutgers University, The State University of New Jersey, Piscataway, NJ, United States

**Keywords:** hippocampal neurons, dendrite arborization, brain-derived neurotrophic factor, Sholl analysis, mRNA, 3′ UTR

## Abstract

The patterning of dendrites is regulated by many factors, such as brain-derived neurotrophic factor (BDNF), which our laboratory has previously shown alters the dendritic arbor uniquely depending on the mode of extracellular application. In the current work, we examine how BDNF affects dendritogenesis in hippocampal neurons when it is overexpressed intracellularly by transcripts previously reported to be transported to distinct cellular compartments. The *BDNF* gene is processed at two different polyadenylation sites, leading to mRNA transcription with two different length 3′ untranslated regions (UTRs), and therefore, different mRNA localization preferences. We found that overexpression of BDNF mRNA with or without 3′ UTRs significantly alters dendritic branching compared to branching in control neurons as analyzed by Sholl distribution curves. Unexpectedly, we found that the overexpression of the shorter BDNF mRNA (reported to be preferentially targeted to the cell body) results in similar changes to Sholl curves compared to overexpression of the longer BDNF mRNA (reported to be preferentially targeted to both the cell body and dendrites). We also investigated whether the BDNF receptor TrkB mediates these changes and found that inhibiting TrkB blocks increases in Sholl curves, although at different distances depending on the transcript’s UTR. Finally, although it is not found in nature, we also examined the effects of overexpressing BDNF mRNA with the unique portion of the longer 3′ UTR since it was previously shown to be necessary for dendritic targeting of mRNA. We found that its overexpression increases Sholl curves at distances close to the cell body and that these changes also depend on TrkB activity. This work illustrates how the mRNA spatial code affects how BDNF alters local dendritogenesis and how TrkB may mediate these effects. Finally, our findings emphasize the importance of intracellular transport of BDNF mRNAs in the regulation of dendrite morphology.

## Introduction

The overall shape of the dendritic arbor determines the inputs that a neuron receives and how information is interpreted, thus affecting synaptic output (Miller and Jacobs, 1984). Brain-derived neurotrophic factor (BDNF) is a well-known regulator of dendrite morphology (McAllister et al., 1995, 1996; Baker et al., 1998; Horch et al., 1999; Jin et al., 2003; Segal, 2003; Dijkhuizen and Ghosh, 2005; Choi et al., 2009; Gao et al., 2009; Kuczewski et al., 2009; Alder et al., 2012; Lazo et al., 2013; Park and Poo, 2013). Previously, our laboratory reported that BDNF alters the dendritic arbor uniquely depending on how it is applied (Kwon et al., 2011; O’Neill et al., 2017). In this work, we extend our previous studies by probing how overexpression and intracellular transport and targeting of BDNF mRNA affect dendritogenesis during the active branching period (Cline, 2001; Wong and Ghosh, 2002; Charych et al., 2006).

The *BDNF* gene is transcribed from nine different promoters (Metsis et al., 1993; Timmusk et al., 1993) and can be processed at two different polyadenylation sites, resulting in transcription of BDNF mRNA with a shorter 3′ untranslated region (UTR; termed “Short” in this study) and with a longer 3′ UTR (termed “Short & Long” in this study since it includes the same initial region as in “Short”) (Liu et al., 2005, 2006; Aid et al., 2007). Both transcripts are present in different relative amounts in various brain regions, and they are preferentially localized to different subcellular compartments while the neuron is at rest. The shorter 3′ UTR has been reported to preferentially target BDNF transcripts to the soma (An et al., 2008) while the longer 3′ UTR has been reported to preferentially target transcripts to both the soma and dendrites (An et al., 2008; Vicario et al., 2015). Stimulating neurons with 10 mM KCl results in BDNF mRNA being targeted to distal dendrites regardless of which 3′ UTR is present (Baj et al., 2011; Vicario et al., 2015). However, the mechanisms responsible for KCl-induced translocation of the mRNAs differ depending on which UTR is present: transport of mRNA with the shorter 3′ UTR is regulated by NT-3, whereas transport of mRNA with the longer 3′ UTR is regulated by exposure to BDNF. Moreover, the mRNAs rely on different sets of RNA-binding proteins for these unique mechanisms (Oe and Yoneda, 2010; Vicario et al., 2015).

The shorter 3′ UTR also plays important roles in other aspects of neuronal physiology. In particular, specific regions within the shorter 3′ UTR are necessary for activity-dependent stabilization of the mRNA caused by calcium influx (Fukuchi and Tsuda, 2010). The shorter 3′ UTR also mediates translation at basal activity levels (Lau et al., 2010; Vaghi et al., 2014). In contrast, the longer 3′ UTR acts as a translational suppressor at basal activity levels but as a translational enhancer, via a HuD/PKC-dependent mechanism, when the neuron is active (Lau et al., 2010; Vaghi et al., 2014; Vanevski and Xu, 2015). Furthermore, translation of transcripts containing the shorter 3′ UTR increases phosphorylation of TrkB, CREB, and other proteins that lead to enhanced synaptic plasticity, and the *in vivo* consequences of this translation improve both short- and long-term memory formation (Wang et al., 2016).

The longer 3′ UTR also plays important roles in physiology. In mutant mice expressing BDNF mRNA containing only the shorter 3′ UTR, targeting of BDNF transcripts to hippocampal dendrites is severely impaired although total BDNF mRNA and protein levels are not affected. This difference in localization does not affect hippocampal dendrite structure, but spines on distal dendrites are thinner and more numerous due to insufficient pruning and lack of maturation, leading to an impairment of long-term potentiation (An et al., 2008). Interestingly, in a different study using the same mice, induction of epileptic seizures with pilocarpine results in transport of BDNF mRNA in both knockout and wild type mice (Vicario et al., 2015), confirming that transport of BDNF mRNA differs significantly depending on whether the neuron is in an active or resting state. Finally, mice lacking expression of the longer 3′ UTR become obese, and injection of a virus encoding BDNF with the longer 3′ UTR completely rescues the phenotype, indicating that the longer 3′ UTR plays a role in energy balance (Liao et al., 2012).

In addition to studying the effects of overexpressing BDNF mRNAs with the shorter and longer 3′ UTRs, we have also chosen to study BDNF mRNA containing the sequences unique to the longer 3′ UTR even though this transcript is not found in nature (called “Long” in this study to distinguish it from “Short & Long”). There is conflicting evidence in the literature as to whether the “Long” 3′ UTR is capable of targeting mRNA to the distal regions of dendrites, with some groups finding enhanced targeting (An et al., 2008) and others finding no difference from the control (Oe and Yoneda, 2010). The goal of our work is to add to the existing body of literature by directly comparing the effects on dendrites of overexpressing BDNF mRNA with the “Long” 3′ UTR to those of overexpressing mRNA with the “Short” and “Short & Long” 3′ UTRs.

This work explores the changes that occur to the dendritic arbor of hippocampal neurons as a result of overexpressing BDNF transcripts containing different length 3′ UTRs. We overexpressed BDNF mRNA from day *in vitro* (DIV) 7–10 as we had done previously for extracellular BDNF application experiments (Kwon et al., 2011; O’Neill et al., 2017), and we consider the results of this study in context with our previous work. We found that the 3′ UTR, and hence intracellular transport and targeting, of BDNF mRNA affects the spatial arrangement of and the number and length of dendrites. Moreover, we treated neuronal cultures with the TrkB inhibitor ANA-12 to determine whether TrkB signaling is involved in these changes to the arbor. Our results suggest that transport and targeting of BDNF mRNA and TrkB signaling coordinate to shape dendrites.

## Materials and Methods

### Primary Culture of Hippocampal Neurons

Hippocampal neurons were isolated from embryonic rats at day 18 of gestation (E18) as previously described (Firestein et al., 1999). After isolation, the hippocampi were dissociated via manual trituration and plated at a density of 2 × 10^5^/well on 12-mm glass coverslips (Fisher) in 24 well plates (Corning). Coverslips were coated with 0.5 mg/ml poly-D-lysine (PDL; Sigma) for at least 1 h at 37°C prior to plating cells. Cultures were maintained in Neurobasal medium supplemented with B27, GlutaMAX, and penicillin/streptomycin (all from Life Technologies) in a humidified 37°C incubator with 5% CO_2_. Cells were grown for 7 days *in vitro* (DIV) prior to transfection. This study was carried out in accordance with the recommendations of the Rutgers University Institutional Animal Care and Use Committee (IACUC). The protocol was approved by the Rutgers University IACUC.

### Plasmid Construction

To generate the plasmids used in this study, the BDNF cDNA fragment containing the coding sequence and longer 3′ UTR (nucleotides 661–4252 of GenBank accession number NM_012513) was generated by performing reverse transcription polymerase chain reaction (RT-PCR) using mRNA from the hippocampi of Sprague Dawley rats (Oe and Yoneda, 2010). The BDNF coding sequence (BDNF cds) was subcloned into the pEGFP-C1 vector between the XhoI and EcoRI restriction sites. The BDNF coding sequence with the shorter 3′ UTR (“Short”; 3′ nucleotides 1–321), with the longer 3′ UTR (“Short & Long”; 3′ nucleotides 1–2842), and with the region unique to the longer 3′ UTR (“Long”; 3′ nucleotides 322–2842) were subcloned into pEGFP-C1 between the XhoI and KpnI restriction sites. PCR was used to isolate Long by inserting an EcoRI restriction site after the BDNF coding sequence and before the beginning of the 3′ UTR. The forward primer containing the XhoI restriction site that was used for all constructs was the following: 5′-CCCCCTCGAGAAATGACCATCCTTTTCCTTA CTA-3′. The reverse primer containing the EcoRI restriction site that was used to generate pEGFP-C1-BDNF cds was the following: 5′-CCCCGAATTCCTATCTTCCCCTTTTAATGGTC GTCAGT-3′. The reverse primer containing the KpnI restriction site that was used to generate pEGFP-C1-BDNF cds + Short 3′ UTR was the following: 5′-CCCCGGTACCGTTTAT TATCAATTCACAATTAAAGCA-3′. The reverse primer containing the KpnI restriction site that was used to generate both pEGFP-C1-BDNF cds + Long 3′ UTR and pEGFP-C1-BDNF cds + Short & Long 3′ UTR was the following: 5′-CCCCGGTACCATTTAAAACATATATTATATGT TAATTGGTACACT-3′. The forward primer used to isolate the unique region of the longer 3′ UTR and add an EcoRI restriction site was the following: 5′-CCCCGAATTCATGTCC CTCTTTCAGAAAACAGACA-3′. The 3′ nucleotide numbers are in reference to those used in Oe and Yoneda (2010).

Initial experiments attempted to use these pEGFP-C1 constructs, with and without co-transfection with mOrange, but the expression of GFP was too dim to reliably determine which neurons were transfected. Therefore, we subcloned BDNF, Short, Long, and Short & Long into the first multiple cloning site (MCS A) of pIRES2-EGFP (Clontech). We first digested the pEGF-C1 constructs and the pIRES2-EGFP vector with the appropriate restriction enzymes, and then we ligated vector (digested pIRES2-EGFP) and insert (digested BDNF fragments). The new BDNF construct contains rat BDNF cds between the XhoI and EcoRI restriction sites of pIRES2-EGFP. The new Short, Long, and Short & Long constructs, respectively, contain rat BDNF cds + Short, rat BDNF cds + Long, and rat BDNF cds + Short & Long between the XhoI and SacII restriction sites of pIRES2-EGFP. We used the pIRES2-EGFP vector because it contains an internal ribosomal entry site after the first MCS and before EGFP. This results in transcription of the BDNF and GFP mRNAs together but translation of the BDNF and GFP proteins separately, allowing for better visualization of GFP expression. Finally, we used the QuikChange II Site-Directed Mutagenesis Kit (Agilent) to introduce a Kozak sequence at the start of the BDNF coding sequence of all constructs to enhance expression. The forward and reverse primers, respectively, used for site-directed mutagenesis were the following: 5′-CGGACTCAGATCTCGAGACCATGACCATCC-3′ and 5′-GGATGGTCATGGTCTCGAGATCTGAGTCCG-3′. Our multiple sequencing results showed that there is an additional T at 3′ nucleotide 1004 in Long and Short & Long.

### BDNF Protein Expression Analysis

**FIGURE 1:**
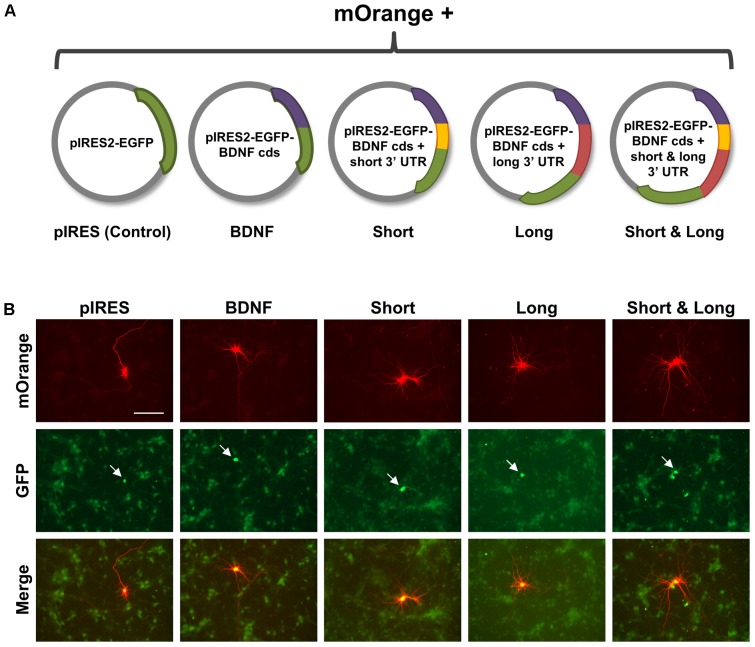
Experimental setup for comparing effects of overexpressing brain-derived neurotrophic factor (BDNF) transcripts on hippocampal neurons. **(A)** Schematic of constructs used in this work. Control is pIRES2-EGFP, with the GFP sequence represented as a green arc. BDNF is pIRES2-EGFP containing the BDNF coding sequence (cds) without targeting UTRs, represented as a purple arc. Short is pIRES2-EGFP containing BDNF cds with the shorter 3′ UTR, represented as purple and yellow arcs, respectively. Long is pIRES2-EGFP containing the BDNF cds (purple arc) with the region of the longer 3′ UTR that is unique (red arc). Short & Long is pIRES2-EGFP containing the BDNF cds (purple arc) and the entire longer 3′ UTR, which is represented by both a yellow arc and a red arc. The IRES site occurs prior to the beginning of the GFP coding sequence. **(B)** Representative images for each condition. Neurons co-expressing mOrange and GFP are indicated with white arrows. Scale bar = 100 μm.

To compare expression levels of the different BDNF transcripts, HEK293T cells were plated at a density of 1 × 10^6^ per well in a 6-well plate and transfected at 24 h after plating with the appropriate plasmid constructs (**Figure 1A**) using Lipofectamine 2000 (Invitrogen) per manufacturer’s instructions. Cultures were scraped and lysed at 48 h after transfection in 1x RIPA buffer (50 mM Tris-HCl, pH 7.4; 150 mM NaCl; 0.5% deoxycholate; 1% NP-40; 1 mM EDTA, pH 7.4; 0.1% SDS) containing 1 mM phenylmethylsulfonyl fluoride (PMSF) and 1x Protease Inhibitor Cocktail (Roche). Lysates were passed through 20 and 25 gauge needles (10 times each), placed on a nutator for 1 h, and centrifuged at 12000 ×*g* for 15 min at 4°C. Protein concentrations of lysate supernatant fractions were determined using the Pierce BCA protein assay kit (ThermoFisher). Proteins (30 μg per condition) were resolved on 15% SDS-polyacrylamide gels and transferred to PVDF membranes. Membranes were blocked with 5% bovine serum albumin (BSA) in TBST (20 mM Tris, pH 7.5; 150 mM NaCl; 0.1% Tween-20) for 1 h. Membranes were probed for 1 h with the following primary antibodies diluted in 3% BSA in TBST: mouse monoclonal anti-BDNF (1:500; Abcam; cat. # ab203573) and mouse monoclonal anti-GAPDH (1:1000; EMD Millipore; cat. # MAB374). Membranes were washed with TBST and probed with mouse IgG (H&L) secondary antibody peroxidase conjugated (1:5000; Rockland, Inc.; cat. # 610-1319) diluted in 5% non-fat dry milk in TBST. Protein bands were visualized with HyGLO Quick Spray Chemiluminescent HRP Antibody Detection Reagent (Denville Scientific, Inc.). Blots were scanned, and band intensities quantified using ImageJ software (NIH). Two independent experiments were performed and showed no difference in expression levels of BDNF transcripts (Supplementary Figure 1).

### Optimization of Transfection Procedure

While transfection using pIRES2-EGFP constructs allows for identification of transfected neurons due to improved GFP fluorescence, the fluorescence is dim and does not extend far enough into the dendrites to allow for sufficiently detailed tracing due to high background. Therefore, co-transfection with a plasmid encoding mOrange was performed using a mass ratios of 1:4 (pmOrange:pIRES). This ratio of pmOrange:pIRES that results in the brightest transfection and highest co-transfection rate is 1:4 and used for all experiments.

### Primary Neuronal Transfection and Treatment With DMSO or ANA-12

Hippocampal neurons were co-transfected at DIV 7 with pmOrange and one of the following plasmids as shown in **Figure 1A**: pIRES2-EGFP (control; “pIRES”), pIRES2-EGFP-BDNF cds (coding sequence; “BDNF”), pIRES2-EGFP BDNF cds + short 3′ UTR (“Short”), pIRES2-EGFP-BDNF cds + long 3′ UTR (“Long”), pIRES2-EGFP-BDNF cds + short & long 3′ UTR (“Short & Long”). Effectene (Qiagen) was used as the transfection reagent according to the manufacturer’s instructions. At 16 h after transfection, hippocampal cultures were treated with either 0.1% DMSO (vehicle) or ANA-12 (10 μM; Tocris). A concentration of 10 μM for ANA-12 treatment was chosen based on previous work and to ensure full antagonism of TrkB (Cazorla et al., 2011; Alder et al., 2013).

### Immunostaining

Neurons were fixed at DIV 10, approximately 72 h after transfection, in 4% paraformaldehyde in phosphate-buffered saline (PBS) for 15 min at room temperature (RT). After fixing, coverslips were washed three times in PBS and incubated in blocking buffer (0.1% Triton X-100, 0.02% sodium azide, 2% normal goat serum in PBS) for 1 h at RT. Coverslips were incubated in primary antibodies [chicken anti-GFP (1:500; ThermoFisher, cat. # PA1-9533) and mouse anti-MAP2 (1:1000; BD Biosciences, cat. # 556320)] diluted in blocking buffer overnight at 4°C. After primary antibody incubation, coverslips were washed three times with PBS, after which they were incubated with secondary antibodies [Alexa Fluor 488 goat anti-chicken IgY (1:250; ThermoFisher, cat. # A-11039) and Alexa Fluor 647 donkey anti-mouse IgG (1:250; ThermoFisher, cat. # A-31571)] diluted in blocking buffer for 1 h at RT. Staining for mOrange was not necessary due to inherently high fluorescence. After secondary antibody incubation, coverslips were washed twice with PBS and incubated with Hoechst 33342 for 5 min at RT to stain nuclei. Coverslips were washed one final time with PBS and mounted onto glass microscope slides with Fluoromount G (Southern Biotechnology).

### Imaging and Fluorescence Quantification

Transfected cells were visualized by immunofluorescence on an EVOS FL microscope (Thermo Fisher Scientific) using a 20x objective. Neurons were initially identified by mOrange expression under the RFP channel, and co-transfection was confirmed by GFP expression. Images of mOrange, GFP, and DAPI fluorescence were taken for each neuron. After imaging and before dendrite tracing, GFP and mOrange fluorescence were quantified using the corrected total cell fluorescence (CTCF) method. A CTCF threshold for GFP fluorescence was set for neurons determined to co-express mOrange and GFP by eye, and all neurons falling below this threshold were excluded from further analysis. mOrange fluorescence was quantified but not used for confirming co-transfection.

### Assessment of Dendrite Number Using Semi-automated Sholl Analysis

Dendrites were assessed as previously described (Kutzing et al., 2010; Langhammer et al., 2010; Kwon et al., 2011; O’Neill et al., 2017) using the most common Sholl analysis, the Inside-Out labeling scheme (O’Neill et al., 2015). We used the “Bonfire” program developed by our laboratory to perform semi-automated Sholl analysis with a 6 μm ring interval starting at 0 μm from the soma. The experimenter was blinded to conditions during all data analyses. Dendrites less than 3 μm in length were not counted (Yu and Malenka, 2004; Charych et al., 2006). Experiments were repeated three to five times.

Prism (Graphpad) was used for determining significance by two-way ANOVA followed by Bonferroni’s or Tukey’s multiple comparisons test for Sholl curves. InStat (GraphPad) was used for determining significance by one-way ANOVA or Kruskal–Wallis test (non-parametric ANOVA) followed by the appropriate multiple comparisons test as noted for dendrite numbers and lengths.

## Results

### Targeting of BDNF Transcripts Affects Spatial Distribution of the Dendritic Arbor

Previous work from our laboratory examined the mechanism by which extracellular application of BDNF shapes the dendritic arbor, either via bath application (Kwon et al., 2011) or via delivery on microbeads (O’Neill et al., 2017). To understand how development of the dendritic arbor is affected by intracellular overexpression and targeting of BDNF mRNA, hippocampal cultures were transfected at DIV 7 with pIRES or plasmids encoding BDNF cds (coding sequence only), Short (BDNF mRNA with the shorter 3′ UTR), Long (BDNF mRNA with the unique region of the longer 3′ UTR), or Short & Long (BDNF mRNA with the full longer 3′ UTR) and fixed at DIV 10 (**Figures 1A,B**). Importantly, this time window matches the 72 h treatment time in our previous studies on the effects of global application (Kwon et al., 2011) and microbead delivery (O’Neill et al., 2017) of BDNF on the dendritic arbor.

**FIGURE 2:**
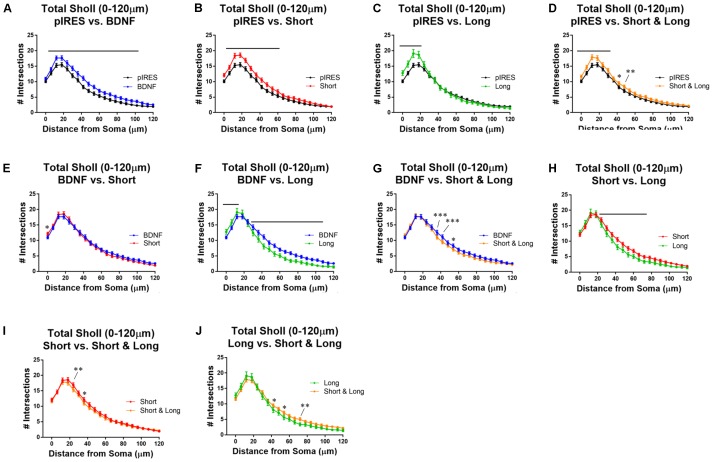
Overexpression of BDNF transcripts results in changes to total Sholl curves. Stars indicate level of significance, and bars indicate significance of at least *p* < 0.05. **(A)** Neurons that express BDNF cds have significantly more dendrites than control neurons (pIRES) at 6–102 μm from the soma. **(B)** Neurons expressing Short show significantly more dendrites than control neurons at 6–60 μm from the soma. **(C)** Neurons expressing Long show significantly more dendrites than control neurons at 0–18 μm from the soma. **(D)** Neurons expressing Short & Long show significantly more dendrites than control neurons at 0–30 and 42–48 μm from the soma. **(E)** Neurons expressing Short show significantly more dendrites than neurons expressing BDNF cds at the soma. **(F)** Neurons expressing Long show significantly more dendrites than neurons expressing BDNF cds at 0–12 μm and significantly fewer dendrites at 30–108 μm from the soma. **(G)** Neurons expressing Short & Long show significantly fewer dendrites than neurons expressing BDNF cds at 36–42 and 54 μm from the soma. **(H)** Neurons expressing Long show significantly fewer dendrites than neurons expressing Short at 24–72 μm from the soma. **(I)** Neurons expressing Short & Long show significantly fewer dendrites than neurons expressing Short at 24 and 36 μm from the soma. **(J)** Neurons expressing Short & Long show significantly more dendrites than neurons expressing Long at 42, 54, and 72 μm from the soma. Error bars indicate SEM. Statistics calculated by two-way ANOVA followed by Bonferroni’s multiple comparisons test (^∗^*p* < 0.05, ^∗∗^*p* < 0.01, ^∗∗∗^*p* < 0.001). n(pIRES) = 68; n(BDNF) = 74; n(Short) = 76; n(Long) = 33; n(Short & Long) = 79.

To determine how dendrite numbers are affected in a distance-dependent manner, we performed Sholl analysis for all orders of dendrites (Total Sholl; **Figure 2**) (Langhammer et al., 2010; O’Neill et al., 2015). Importantly, a significant increase in dendrites occurs regardless of which BDNF transcript is expressed. All conditions are compared to each other in **Figure 2**, and the statistics are summarized in **Table 1**.

**Table 1:** Statistical details of comparisons between conditions for Total Sholl analysis in **Figure 2**.

Total Sholl	Locations of significance (μm)	Level	Effect
**pIRES vs. BDNF**	6–102	^∗^-^∗∗∗∗^	+
**pIRES vs. Short**	6–60	^∗∗^-^∗∗∗∗^	+
**pIRES vs. Long**	0–18	^∗∗∗∗^	+
**pIRES vs. Short & Long**	0–30, 42–48	^∗^-^∗∗∗∗^	+
**BDNF vs. Short**	0	^∗^	+
**BDNF vs. Long**	0–12, 30–108	^∗^-^∗∗∗∗^	+, -
**BDNF vs. Short & Long**	36–42, 54	^∗∗∗^, ^∗^	-
**Short vs. Long**	24–72	^∗^-^∗∗∗∗^	-
**Short vs. Short & Long**	24, 36	^∗∗^, ^∗^	-
**Long vs. Short & Long**	42, 54, 72	^∗^, ^∗^, ^∗∗^	+

*The table shows sites of significant differences in Sholl curves (μm from the soma), the level of significance, and the effect of significance (whether increased or decreased dendrites). A positive (“+”) effect for pIRES vs. BDNF means that neurons expressing BDNF show significantly more dendrites than control neurons expressing pIRES at indicated distances from the soma. Statistics calculated by two-way ANOVA followed by Bonferroni’s multiple comparisons test (^∗^p < 0.05, ^∗∗^p < 0.01, ^∗∗∗^p < 0.001, ^∗∗∗∗^p < 0.0001).*

All neurons transfected with BDNF plasmids have significantly more dendrites than control neurons, whether or not 3′ UTRs are present (**Figures 2A–D**). Interestingly, expression of BDNF cds results in significantly increased dendrites over the largest area (6–102 μm from the soma; **Figure 2A**). Expression of the Short construct, which is thought to preferentially target BDNF mRNA to the soma, results in increased dendrites at 6–60 μm from the soma (**Figure 2B**). This increase occurs at fewer sites in the arbor than in neurons transfected with BDNF cds construct (**Figure 2A** vs. **Figure 2B**). Transfection of neurons with the Long construct results in increased dendrites at 0–18 μm from the soma (**Figure 2C**). Notably, this increase occurs at fewer sites in the arbor than in neurons transfected with constructs for BDNF or Short. Finally, transfection of neurons with the Short & Long construct, which is thought to target BDNF mRNA to both the soma and dendrites, significantly increases dendrites at 0–30 and 42–48 μm from the soma (**Figure 2D**). Significant changes to the arbor in neurons expressing Short & Long occur at distances intermediate of neurons expressing Short and those expressing Long.

To understand how the targeting of BDNF mRNA affects the arbor when BDNF is overexpressed, we compared neurons transfected with constructs for BDNF cds to those transfected with constructs for BDNF with one of the 3′ UTRs (**Figures 2E–G**). Neurons expressing BDNF cds and neurons expressing Short exhibit similar Sholl curves (**Figure 2E**), but neurons expressing Short show increased dendrites at the soma. Neurons expressing Long exhibit distinct Sholl curves from neurons expressing BDNF cds. Neurons expressing Long show significantly more dendrites proximal to the soma (0–12 μm from the soma), but neurons expressing BDNF show significantly more dendrites distal to the soma (30–108 μm from the soma; **Figure 2F**). Neurons expressing Short & Long show significantly fewer dendrites at 36, 42, and 54 μm from the soma when compared with neurons expressing BDNF cds (**Figure 2G**).

Finally, we compared the effects of overexpressing BDNF mRNA with different 3′ UTRs (**Figures 2H–J**). Neurons expressing Short show significantly more dendrites than neurons expressing Long at 24–72 μm from the soma (**Figure 2H**). Neurons expressing Short also show significantly more dendrites than neurons expressing Short & Long, but only at two sites (24 and 36 μm from the soma; **Figure 2I**). Finally, neurons expressing Long show significantly fewer dendrites than neurons expressing Short & Long at 42, 54, and 72 μm from the soma (**Figure 2J**).

These comparisons illustrate that targeting BDNF mRNA with the different 3′ UTRs exerts distinct effects on the overall dendritic arbor. Surprisingly, overexpression of Short results in very similar changes to the dendritic arbor when compared to overexpression of Short & Long although evidence exists supporting distinct localization preferences for mRNAs with either of these two UTRs (An et al., 2008). Additionally, the data show that overexpression of Short & Long results in an intermediate effect observed when either mRNA with solely Short or Long is expressed. Finally, we found it surprising that overexpression of Long changes proximal but not distal dendrites.

### Targeting of BDNF Transcripts Affects the Number and Length of Specific Orders of Dendrites

**FIGURE 3:**
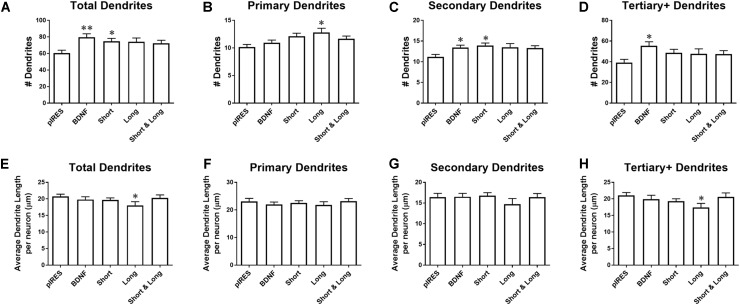
Overexpression of BDNF mRNA has distinct effects on specific orders of dendrites. **(A)** Neurons expressing BDNF and Short have significantly more dendrites than control neurons (pIRES). **(B)** Neurons expressing Long have significantly more primary dendrites than control neurons. **(C)** Neurons expressing BDNF and Short have significantly more secondary dendrites than control neurons. **(D)** Neurons expressing BDNF have significantly more tertiary and higher order dendrites than control neurons. **(E)** Neurons expressing Long have significantly shorter dendrites than control neurons. **(F)** Primary dendrite length does not significantly change as a result of transfection or treatment for any condition. **(G)** Secondary dendrite length does not significantly change as a result of transfection or treatment for any condition. Four outliers were eliminated from the BDNF condition using the ROUT method with Q = 1%. **(H)** Neurons expressing Long show significantly shorter tertiary and higher order dendrites than control neurons. Error bars indicate SEM. Statistics calculated using the Kruskal–Wallis test followed by Dunn’s multiple comparisons test (^∗^*p* < 0.05, ^∗∗^*p* < 0.01). All conditions were compared to neurons transfected with pIRES (control) and to neurons expressing BDNF. n(pIRES) = 68; n(BDNF) = 74; n(Short) = 76; n(Long) = 33; n(Short & Long) = 79.

We investigated the effects of overexpressing BDNF transcripts on the number and length of dendrites. Neurons transfected with constructs for BDNF cds or Short have significantly more total dendrites than control neurons (pIRES; **Figure 3A**). Neurons expressing Long show significantly more primary dendrites than do control neurons (**Figure 3B**), and neurons expressing BDNF cds or Short show significantly more secondary dendrites than control neurons (**Figure 3C**). Neurons expressing BDNF cds show significantly more tertiary and higher order dendrites than control neurons (**Figure 3D**). For neurons expressing Short & Long, the number of dendrites does not differ significantly compared to control neurons for any category of dendrite.

Overexpression of BDNF mRNA causes more subtle changes to the lengths of various orders of dendrites. Neurons expressing Long show significantly shorter tertiary and higher order dendrites than control neurons (**Figure 3E**). Overexpression of BDNF, Short, or Short & Long does not significantly change the length of any order of dendrite compared to control neurons. Additionally, primary and secondary dendrite lengths are unaffected by overexpression of any BDNF mRNA (**Figures 3F,G**). Finally, neurons expressing Long show significantly shorter tertiary and higher order dendrites than control neurons (**Figure 3H**).

Based on the results from **Figure 2**, we had expected the constructs that caused the greatest changes to Sholl curves (BDNF, Short, and Short & Long) to also significantly change dendrite numbers in one or more categories (**Figure 3**). This hypothesis was partially confirmed by BDNF and Short but not by Short & Long, indicating that changes to Sholl curves caused by BDNF or by Short are order specific (due to secondary and higher order dendrites) but that changes caused by Short & Long are not order specific. Additionally, when comparing the results in **Figure 2** to those in **Figure 3**, the changes to the proximal area of the Sholl curve for Long are likely due to increases in primary dendrites. Interestingly, Long is the only construct that causes any changes to dendrite length; perhaps the addition of primary dendrites depletes a limiting reagent that then results in shorter tertiary and higher order dendrites. Finally, the lack of change to dendrite length after overexpression of the other constructs suggests that there might not be a limiting reagent in those cases since, when dendrites are added, the lengths are not significantly different.

### TrkB Partially Modulates Changes to the Overall Dendritic Arbor by BDNF Transcripts

**FIGURE 4:**
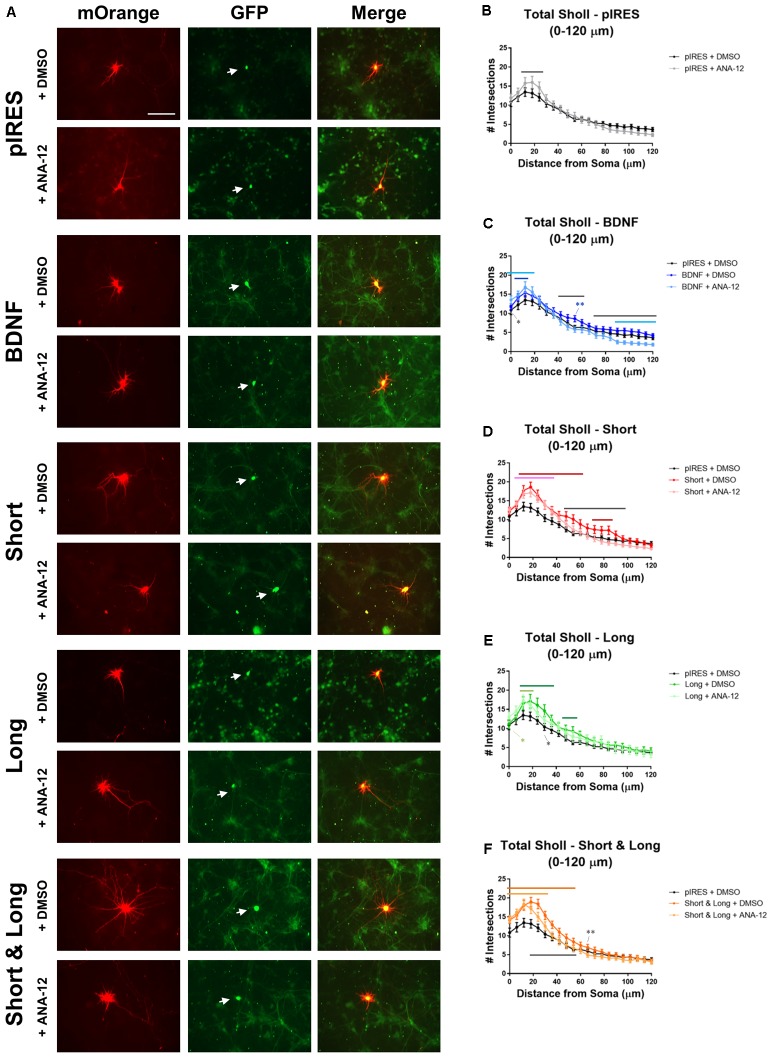
TrkB activity plays a role in BDNF-mediated changes to the dendritic arbor. Stars indicate level of significance, and bars indicate significance of at least *p* < 0.05. Black bars indicate significant differences between vehicle (DMSO) and ANA-12 treatment of the same condition; dark colored bars indicate significant differences between the condition + DMSO and pIRES + DMSO; light colored bars indicate significant differences between the condition + ANA-12 and pIRES + DMSO. **(A)** Representative images for each condition. Neurons co-expressing mOrange and GFP are indicated with white arrows. Scale bar = 100 μm. **(B)** Total Sholl analysis of the first 120 μm for neurons overexpressing pIRES. **(C)** Total Sholl analysis of the first 120 μm for neurons overexpressing BDNF cds. **(D)** Total Sholl analysis of the first 120 μm for neurons overexpressing Short. **(E)** Total Sholl analysis of the first 120 μm for neurons overexpressing Long. **(F)** Total Sholl analysis of the first 120 μm for neurons overexpressing Short & Long. Error bars indicate SEM. Statistics calculated by two-way ANOVA followed by Tukey’s multiple comparisons test (^∗^*p* < 0.05, ^∗∗^*p* < 0.01). n(pIRES + DMSO) = 20; n(BDNF + DMSO) = 36; n(Short + DMSO) = 28; n(Long + DMSO) = 16; n(Short & Long + DMSO) = 32; n(pIRES + ANA-12) = 23; n(BDNF + ANA-12) = 31; n(Short + ANA-12) = 34; n(Long + ANA-12) = 18; n(Short & Long + ANA-12) = 28.

TrkB receptors are expressed throughout cultured hippocampal dendrites as are truncated versions of this receptor (Kryl et al., 1999). Overexpression of full length TrkB (TrkB.FL) increases proximal dendrites with a concomitant decrease in more distal dendrites while overexpression of truncated T1 (TrkB.T1) elongates distal dendrites, and the two receptors inhibit each other (Yacoubian and Lo, 2000). Since, the different receptors mediate distinct effects on the arbor, we asked whether TrkB signaling mediates the changes that occur to the dendritic arbor as a result of BDNF mRNA overexpression. We repeated the aforementioned experiments in the presence of TrkB inhibitor ANA-12, following previously established protocols (Cazorla et al., 2011; Alder et al., 2013). Hippocampal cultures were transfected at DIV 7, treated with vehicle or 10 μM ANA-12 at 16 h post-transfection, and fixed at 72 h post-transfection (**Figure 4A**). Sholl analysis was performed for all orders of dendrites (**Figures 4B–F**). We found that inhibiting TrkB blocks some of these changes, and a summary of statistically significant differences is shown in **Table 2**. In this set of experiments, control neurons are those expressing pIRES and treated with DMSO (pIRES + DMSO).

**Table 2:** Statistical details of comparisons between conditions for Total Sholl analysis in **Figure 4**.

Total Sholl	Locations of significance (μm)	Level	Effect
**plRES + DMSO vs. plRES + ANA-12**	12–24	^∗∗^-^∗∗∗^	+
**plRES + DMSO vs. BDNF + DMSO**	6–12, 54	^∗^, ^∗∗^	+
**pIRES + DMSO vs. BDNF + ANA-12**	0–18, 90–132, 150–156	^∗^-^∗∗∗∗^	+, -, -
**BDNF + DMSO vs. BDNF + ANA-12**	0, 42–60, 72–174	^∗^-^∗∗∗∗^	+, -, -
**plRES + DMSO vs. Short + DMSO**	12–60, 72–84	^∗^-^∗∗∗∗^	+
**pIRES + DMSO vs. Short + ANA-12**	6–36, 150–156	^∗^-^∗∗∗∗^	+, -
**Short + DMSO vs. Short + ANA-12**	48–96	^∗^-^∗∗∗∗^	-
**plRES + DMSO vs. Long + DMSO**	12–36, 48–54	^∗^-^∗∗∗∗^	+
**pIRES + DMSO vs. Long + ANA-12**	0, 12–18	^∗^-^∗∗∗∗^	+
**Long + DMSO vs. Long + ANA-12**	30	^∗^-^∗∗∗∗^	-
**pIRES + DMSO vs. Short & Long + DMSO**	0–54	^∗^-^∗∗∗∗^	+
**pIRES + DMSO vs. Short & Long + ANA-12**	0–30	^∗^-^∗∗∗∗^	+
**Short & Long + DMSO vs. Short & Long + ANA-12**	18–54, 66	^∗^-^∗∗∗∗^	-

*The table shows sites of significant differences in Sholl curves (μm from the soma), the level of significance, and the effect of significance (whether increased or decreased dendrites). For example, a positive (“+”) effect for pIRES + DMSO vs. pIRES + ANA-12 means that neurons expressing pIRES and treated with ANA-12 show significantly more dendrites than neurons expressing pIRES and treated with DMSO (i.e., untreated) at the indicated distances from the soma. Statistics calculated by two-way ANOVA followed by Tukey’s multiple comparisons test (^∗^p < 0.05, ^∗∗^p < 0.01, ^∗∗∗^p < 0.001, ^∗∗∗∗^p < 0.0001).*

In control neurons, treatment with ANA-12 increases dendrites at 12–24 μm from the soma (black bar; **Figure 4B**). Since previous reports have suggested that TrkB.FL and TrkB.T1 inhibit each other (Yacoubian and Lo, 2000) and that TrkB.T1 acts independently of TrkB.FL to increase dendrites (Kryl et al., 1999), it is possible that TrkB.T1 acts on this region of the arbor since TrkB.FL has been inhibited. To understand how inhibition of TrkB.FL via ANA-12 affects Sholl curves after overexpression of the different BDNF transcripts, we then performed the following comparisons: (1) neurons overexpressing the mRNA and treated with DMSO versus the control; (2) neurons overexpressing the mRNA and treated with ANA-12 versus the control; and (3) neurons overexpressing the mRNA and treated with DMSO versus neurons overexpressing the mRNA and treated with ANA-12.

Neurons expressing BDNF cds have significantly increased dendrites close to the cell body compared with control neurons, regardless of whether they were treated with ANA-12 or DMSO (dark blue and light blue bars, respectively; **Figure 4C**). Interestingly, the region of the Sholl curve showing significant increases is larger for neurons treated with ANA-12 (0–18 μm from the soma; light blue bar) than for neurons treated with DMSO (6–12 μm from the soma; dark blue bar) when compared to control neurons. Additionally, at the soma (0 μm), neurons expressing BDNF cds and treated with ANA-12 have significantly more dendrites than neurons expressing BDNF cds and treated with DMSO (black star). However, for the ANA-12 treated neurons, this increase in proximal dendrites may occur at the expense of distal dendrites because, in distal regions, these neurons have significantly fewer dendrites compared with control neurons (90–132 and 150–156 μm from the soma; light blue bar) and compared with neurons expressing BDNF and treated with DMSO (42–60 and 72–174 μm from the soma; black bars). These data suggest that TrkB.FL does not play a role in regulating proximal branching but may play a role in regulating distal branching for neurons overexpressing BDNF.

Neurons expressing Short and treated with DMSO have significantly more dendrites than do control neurons at 12–60 and 72–84 μm from the soma (dark red bars; **Figure 4D**). Treatment with ANA-12 attenuates these increases since neurons expressing Short and treated with ANA-12 only show increased dendrites at 6–36 and also show decreased dendrites at 150–156 μm from the soma when compared to control neurons (pink bar; **Figure 4D**). Moreover, neurons expressing Short have significantly more dendrites at several locations (48–96 μm from the soma) when they are treated with DMSO compared to when they are treated with ANA-12 (black bar; **Figure 4D**). This suggests that TrkB plays a role in Short-mediated increases to proximal dendrites since these changes occur over a smaller region when TrkB is inhibited.

Neurons expressing Long and treated with DMSO have significantly more dendrites at 12–36 and 48–54 μm from the soma when compared with control neurons (dark green bars; **Figure 4E**). ANA-12 treatment attenuates Long-promoted increases in dendrites as increases are observed only at 0 and 12–18 μm from the soma when compared with control neurons (light green bar and star; **Figure 4E**). Neurons expressing Long and treated with ANA-12 have fewer dendrites at one location (30 μm) when compared with untreated neurons expressing Long and treated with DMSO (black star; **Figure 4E**). These data suggest that TrkB plays a role in Long-mediated increases to dendrites, specifically between 24 and 54 μm from the soma, since these increases are not present in Long-expressing neurons treated with ANA-12.

Neurons expressing Short & Long and treated with DMSO have significantly increased dendrites at 0–54 μm from the soma compared with control neurons (dark orange bar; **Figure 4F**). Similarly, neurons expressing Short & Long and treated with ANA-12 have significantly increased dendrites compared with control neurons, but these increases occur at fewer locations (at 0–30 μm from the soma; light orange bar; **Figure 4F**). Unsurprisingly, neurons expressing Short & Long and treated with ANA-12 have significantly fewer dendrites than the same neurons treated with DMSO at several locations (at 18–54 and 66 μm from the soma; black bar and star; **Figure 4F**). Similar to the Short and the Long data above, these data also suggest that TrkB plays a role in proximal increases to dendrites, specifically between 36 and 54 μm from the soma, since ANA-12 treatment decreases the region of increased dendrites in neurons expressing Short & Long.

The results from this set of experiments indicate how TrkB.FL may be involved in regulating the dendritic arbor after overexpression of distinct BDNF mRNAs. We found it interesting that inhibition of TrkB by ANA-12 does not appear to alter the changes that occur to Sholl curves after expression of BDNF mRNA (BDNF cds without any UTR) but that it does alter the changes that occur to the proximal region of the Sholl curves when BDNF mRNA is present with any UTR, whether physiological (Short and Short & Long) or not (Long). Finally, overexpression of Short results in similar changes to Sholl curves when compared to overexpression of Short & Long, as in **Figure 2**, despite evidence suggesting that these two transcripts have different localization preferences (An et al., 2008). Additionally, the changes caused by ANA-12 treatment are similar when comparing neurons expressing Short and neurons expressing Short & Long. In both cases (**Figures 4D,F**), there is still an increase in the Sholl curve compared to control neurons, but the increase occurs over a smaller area.

### TrkB Regulates BDNF-Mediated Changes to Dendrite Number But Not Length

**FIGURE 5:**
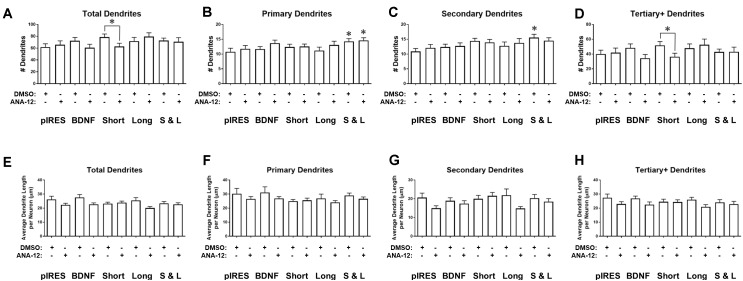
ANA-12 treatment has a minimal effect on BDNF-mediated changes in dendrite number and no effect on dendrite length. **(A)** Neurons expressing Short and treated with ANA-12 have significantly fewer dendrites than neurons expressing Short and treated with DMSO. **(B)** Neurons expressing Short & Long have significantly more dendrites than control neurons, regardless of whether they were treated with DMSO or ANA-12. **(C)** Neurons expressing Short & Long and treated with DMSO have significantly more secondary dendrites than control neurons. **(D)** Neurons expressing Short and treated with ANA-12 have significantly fewer tertiary and higher order dendrites than neurons expressing Short and treated with DMSO. **(E)** Average dendrite length does not significantly change as a result of transfection or treatment for any condition. **(F)** Primary dendrite length does not significantly change as a result of transfection or treatment for any condition. **(G)** Secondary dendrite length does not significantly change as a result of transfection or treatment for any condition. **(H)** Tertiary and higher order dendrite length does not significantly change as a result of transfection or treatment for any condition. Error bars indicate SEM. Statistics calculated by Kruskal–Wallis test followed by Dunn’s multiple comparisons test (^∗^*p* < 0.05). The following comparisons were made for each parameter: pIRES + DMSO vs. condition + DMSO; pIRES + DMSO vs. condition + ANA-12; pIRES + DMSO vs. pIRES + ANA-12; condition + DMSO vs. condition + ANA-12. n(pIRES + DMSO) = 20; n(BDNF + DMSO) = 36; n(Short + DMSO) = 28; n(Long + DMSO) = 16; n(Short & Long + DMSO) = 32; n(pIRES + ANA-12) = 23; n(BDNF + ANA-12) = 31; n(Short + ANA-12) = 34; n(Long + ANA-12) = 18; n(Short & Long + ANA-12) = 28.

We investigated how TrkB signaling shapes the changes that occur to the number and length of dendrites after overexpression of BDNF transcripts. Neurons expressing Short and treated with ANA-12 have significantly fewer total dendrites than neurons expressing Short and treated with DMSO (**Figure 5A**). Neurons expressing Short & Long have significantly more primary dendrites than control neurons, regardless of whether they are treated with ANA-12 or DMSO (**Figure 5B**). These data indicate that TrkB is not involved in these increases in primary dendrites. Untreated neurons expressing Short & Long have significantly more secondary dendrites than control neurons (**Figure 5C**). Therefore, TrkB might be involved in these increases to secondary dendrites since the changes are not significant when Short & Long is expressed and TrkB is inhibited. Neurons expressing Short and treated with ANA-12 have significantly fewer tertiary and higher order dendrites than neurons expressing Short and treated with DMSO (**Figure 5D**). Treatment with ANA-12 does not result in any significant changes in dendrite length of any category (**Figures 5E–H**).

Overall, the changes in dendrite number and length as a result of ANA-12 treatment (**Figure 5**) are more subtle than those seen in Sholl curves (**Figure 4**), which give a picture of the entire arbor shape, after the same treatment. However, combining these results can improve our understanding of the role of TrkB in modulating the dendritic arbor. ANA-12 treatment affects the changes to Sholl curves caused by overexpression of Short, Long, and Short & Long. For neurons overexpressing Short and treated with ANA-12, the increase in the Sholl curve compared to control neurons (**Figure 4D**; pink line) does not appear to be due to changes to a particular category of dendrite. However, the differences between neurons expressing Short and treated with ANA-12 versus neurons expressing Short and treated with DMSO (**Figure 4D**; black line) appear to be due to a significant decrease in tertiary and higher order dendrites after ANA-12 treatment (**Figure 5D**). For neurons overexpressing Long and treated with ANA-12, the changes to the Sholl curves (**Figure 4E**) do not appear to be driven by changes in a specific category of dendrite, which may be the case because Long is not a physiological transcript. For neurons expressing Short & Long and treated with ANA-12, the changes in the Sholl curves compared to control neurons (**Figure 4F**; light orange line) appear to be due to changes in primary dendrites only (**Figure 5B**). On the other hand, when comparing neurons overexpressing Short & Long and treated with ANA-12 to neurons overexpressing Short & Long and treated with DMSO (**Figure 4F**; black line), our data suggest that the differences in Sholl curves are due to secondary dendrites since these are significantly decreased after ANA-12 treatment (**Figure 5C**).

## Discussion

**FIGURE 6:**
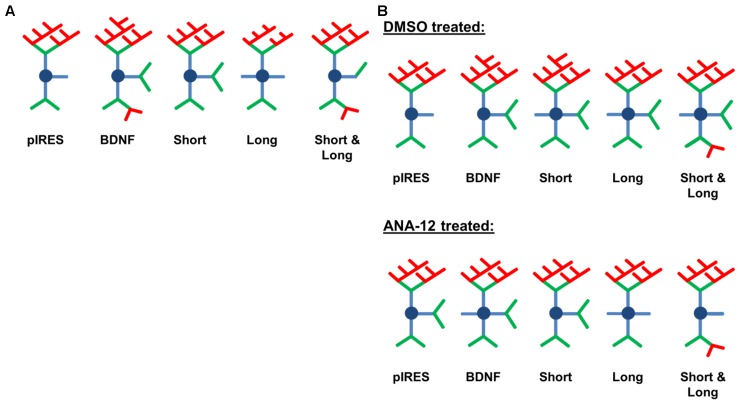
Schematic depicting changes caused by overexpression of BDNF transcripts. Primary dendrites are shown in blue, secondary dendrites in green, and tertiary and higher order dendrites in red. **(A)** Overexpression of BDNF mRNA results in distinct changes to the dendritic arbor that depend on the 3′ UTR. **(B)** ANA-12 treatment has a partial effect on BDNF-mediated shaping of the dendritic arbor.

The work presented here represents the first understanding of how overexpression and 3′ UTR-mediated intracellular transport and targeting of BDNF mRNA, and hence protein, affects the dendritic arbor. We initially hypothesized that overexpression of BDNF would increase dendrite branching and that targeting of BDNF to the soma and to dendrites via mRNA containing the longer 3′ UTR (referred to as Short & Long in this study) would increase branching over a greater range of dendritic distances from the cell body than would targeting of BDNF to only the soma with mRNA containing the shorter 3′ UTR (referred to as Short in this study). Surprisingly, our results suggest the opposite: overexpression of BDNF mRNA with the shorter 3′ UTR increases dendrite branching over a wider range of distances from the soma (**Figure 2B** vs. **Figure 2D**). We also determined the effects of overexpressing the region unique to Short & Long (referred to as Long in this study), which is not a physiological transcript. We hypothesized that overexpression of Long would result in increases to distal dendrites since previous studies had found this region to be sufficient for targeting (An et al., 2008) and that the regions of change would be similar to those seen after overexpression of Short & Long. We again found the opposite to be true: overexpression of Long results in increased dendrites in a small region close to the soma while overexpression of Short & Long results in increases in an intermediate region between Short and Long (**Figure 2C** vs. **Figure 2D**). Additionally, in the second part of our study, we found that TrkB is involved in some of the changes that occur to the arbor as a result of BDNF mRNA overexpression, specifically after overexpression of Short, Long, and Short & Long (**Figures 4D–F**). In particular, significant increases in secondary dendrites as a result of overexpression of Short & Long are prevented when TrkB is inhibited (**Figure 5C**). A schematic summary of the results is shown in **Figure 6**.

We had initially hypothesized that overexpression of BDNF mRNA containing only the coding sequence would result in increases to a small region of the dendritic arbor close to the soma, similar to that which occurs via bath application of BDNF (Kwon et al., 2011). Instead, overexpression of this transcript results in increased dendrites over the widest range of distances from the soma, as observed by conventional Sholl analysis (**Figure 2A**). Overexpression of this transcript also results in the greatest change to dendrite numbers, significantly increasing both secondary and tertiary and higher order dendrites (**Figures 3C,D**). These changes differ when compared to extracellular application of BDNF studied in our previous work, which showed the following: (1) that BDNF significantly increases primary and secondary dendrites proximal to the soma when BDNF is bath administered for 72 h to cultures of hippocampal neurons (Kwon et al., 2011), and (2) that local stimulation of dendrites with BDNF-coated microbeads for 72 h likewise increases primary and secondary dendrites and also prevents pruning of tertiary and higher order dendrites (O’Neill et al., 2017). Neither bath application nor local stimulation with BDNF affect tertiary and higher order dendrite numbers, whereas overexpression of the mRNA containing the BDNF coding sequence alone significantly increases both secondary and tertiary and higher order dendrites (**Figures 3C,D**). Moreover, when examining Sholl curves, increases in the dendritic arbor resulting from BDNF mRNA overexpression not only overlaps with those resulting from bath application of BDNF but are also observed in more distal regions of the arbor, similar to the results from local BDNF stimulation. The effects of BDNF mRNA overexpression on distal dendrites could be due to the role of dendritic targeting elements within the coding sequence, which are mediated by RNA-binding proteins, such as translin (Chiaruttini et al., 2009; Wu et al., 2011). Translin mediates trafficking of BDNF mRNA under basal conditions and after stimulation with 10 mM KCl; thus, BDNF mRNA without a 3′ UTR is not constrained to the soma under active or rest conditions. Our results suggest that the 3′ UTRs of BDNF mRNA are important for BDNF mRNA transport but are not the only necessary aspect for dendritic trafficking.

Targeting of BDNF mRNA influences local translation of the BDNF protein. Dendritically localized BDNF and TrkB mRNAs are not translated unless that portion of the dendrite is stimulated, thus restricting BDNF and TrkB synthesis to synaptically active sites (Bramham and Wells, 2007; Baj et al., 2016). Moreover, it has been proposed that, upon stimulation, BDNF is translated and secreted as pro-BDNF, after which it is cleaved by the tissue plasminogen activator (tPa)/plasmin system extracellularly to form mature BDNF (Waterhouse and Xu, 2009). The newly processed mature BDNF then activates, in an autocrine manner, TrkB receptors at that same synapse (Horch et al., 1999; Waterhouse and Xu, 2009; Harward et al., 2016). It is likely that some or all of the observed effects on dendrite branching caused by overexpression of BDNF mRNA are activity-dependent, and future studies will include determining whether excitatory or inhibitory activity is responsible for the changes. Future work will also attempt to directly correlate how activity in different regions of the neuron (i.e., proximal vs. distal dendrites) is related to changes in dendrite branching. Ideally, live imaging studies would be performed to observe how targeting and transport of BDNF mRNAs influence extension and retraction of dendrites. BDNF has been reported to make dendrites and spines more dynamic (Horch et al., 1999; Horch and Katz, 2002), and it is possible that the different mRNAs uniquely affect the stability of dendrites.

Why do transport and targeting of BDNF mRNA with the shorter (Short) or longer (Short & Long) 3′ UTR result in different changes to the dendritic arbor? It is possible that the differences lie in the fact that the Short mediates translation when the neuron is at rest whereas Short & Long enhances activity-dependent translation (Lau et al., 2010). We also found it interesting that overexpression of the region unique to the longer 3′ UTR (Long) increases dendrites in a small region close to the soma. This may have occurred since Long is not a physiological transcript and this region of the 3′ UTR is not sufficient alone for dendritic targeting (Oe and Yoneda, 2010), which is supported by the fact that neurons overexpressing Short & Long show a larger region of dendritic increase than neurons overexpressing Long. Increased dendritic branching mediated by Long may occur in active regions near the soma where Long promotes translation and increased primary dendrites (Harward et al., 2016). Also, enhancement of at-rest translation by mRNA containing the short 3′ UTR (Lau et al., 2010) may have wider-ranging effects on the dendritic arbor since promotion of translation is not limited to active dendrites.

In the second part of our study, we used a concentration of ANA-12 (10 μM) that is expected to block TrkB.FL activity based on previous studies (Cazorla et al., 2011; Alder et al., 2013); however, we recognize that full inhibition of TrkB may not have occurred. If full inhibition of TrkB did not occur, then the conclusions drawn about the role of TrkB would only be partially correct, and stronger effects may be seen with complete inhibition of the receptor. Additionally, it is interesting that ANA-12 treatment increases proximal dendrites in control neurons. A previous report indicates that TrkB.FL preferentially acts on proximal dendrites (within 35 μm of the soma) while TrkB.T1 acts on more distal dendrites (at distances greater than 40 μm from the soma) (Yacoubian and Lo, 2000). However, since this study was performed using intact slices, the Sholl curve may be shifted slightly when compared to dissociated *in vitro* cultures. Moreover, since the two receptors are shown to inhibit each other in this same study (Yacoubian and Lo, 2000), it is possible that the activity of TrkB.FL is prevented from modifying the proximal region of the arbor after ANA-12 treatment, thus allowing the activity of TrkB.T1 to instead modify this region. The effects of ANA-12 on control cultures could also be due to basal levels of BDNF expression and release by rat hippocampal neurons (Wetmore et al., 1994; Schaaf et al., 1998). It is possible that the differing effects that TrkB inhibition has on neurons overexpressing BDNF mRNAs could be a result of TrkB downregulation caused by overexpression of specific BDNF mRNAs, specifically mRNA with one of the 3′ UTRs.

In summary, this is the first study to examine how the different 3′ UTRs of BDNF mRNA regulate BDNF action on the dendritic arbor. Recently, it was reported that BDNF 5′ UTR transcripts affect the dendritic arbor in hippocampal neurons *in vitro* (Baj et al., 2011) and *in vivo* (Maynard et al., 2017). Thus, the 5′ and 3′ UTRs of BDNF mRNA, as well as regions within the coding sequence, coordinate to transport BDNF mRNA and, thus, shape the dendritic arbor in specific neurons. Our observed results *in vitro* point to the physiological significance of alternative splicing and transport of mRNAs *in vivo*. Our findings also imply that the preferential localization of BDNF transcripts depend on whether the neuron is active or at rest, thereby affecting which region of the dendritic arbor is altered.

## Author Contributions

KO, KD, and AO designed and performed the experiments and prepared the digital images. BF conceived the experiments. All the authors analyzed the data and wrote the article.

## Conflict of Interest Statement

The authors declare that the research was conducted in the absence of any commercial or financial relationships that could be construed as a potential conflict of interest.
