# Supplementary material for: The 3′ UTRs of Brain-Derived Neurotrophic Factor Transcripts Differentially Regulate the Dendritic Arbor
Source: Front Cell Neurosci. 2018 Mar 7;12:60. doi: 10.3389/fncel.2018.00060 (PMC5845904; doi:10.3389/fncel.2018.00060)
Supplement: Supplementary file 1 [file Data_Sheet_1.PDF]

Supplementary Material

The 3' UTRs of Brain-Derived Neurotrophic Factor transcripts differentially regulate the dendritic arbor

Kate M. O'Neill[¥], Katherine E. Donohue[¥], Anton Omelchenko, and Bonnie L. Firestein[*]

*** Correspondence:** Dr. Bonnie L. Firestein, firestein@biology.rutgers.edu

[¥] KMO and KED contributed equally.

1 Supplementary Figures and Tables

1.1 Supplementary Figures

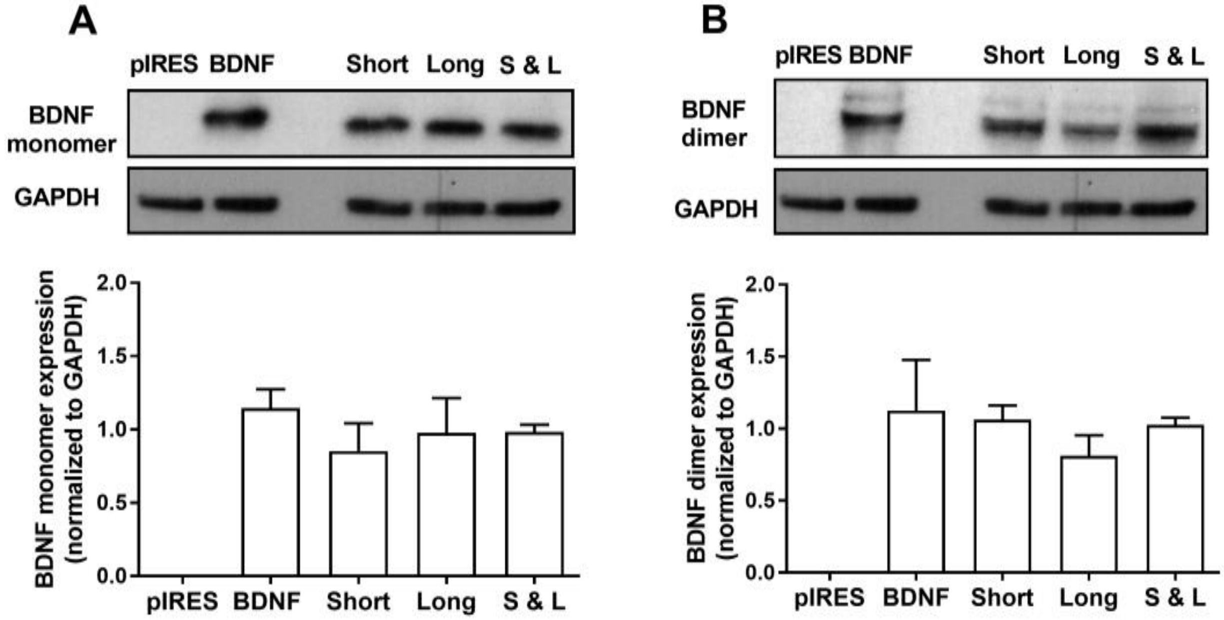

Supplementary Figure 1: Expression levels of BDNF do not differ in HEK cells expressing cDNAs encoding BDNF and BDNF 3' UTRs. A. Western blot analysis of extracts from HEK293T cells transfected with pIRES and constructs for BDNF, Short, Long, and Short & Long demonstrates that there are no significant differences in expression levels of mature BDNF monomers (14 kDa) **B.** Western blot analysis of extracts from HEK293T cells transfected with the same constructs demonstrates that there are no significant difference in expression levels of mature BDNF dimers (28 kDa). Error bars represent SEM. Statistics calculated by one-way ANOVA followed by Tukey's multiple comparisons test. Data were obtained from two independent experiments.
